# Intestinal helminth infection drives carcinogenesis in colitis-associated colon cancer

**DOI:** 10.1371/journal.ppat.1006649

**Published:** 2017-09-22

**Authors:** Eva Pastille, Annika Frede, Henry J. McSorley, Jessica Gräb, Alexandra Adamczyk, Sebastian Kollenda, Wiebke Hansen, Matthias Epple, Jan Buer, Rick M. Maizels, Robert Klopfleisch, Astrid M. Westendorf

**Affiliations:** 1 Institute of Medical Microbiology, University Hospital Essen, University Duisburg-Essen, Essen, Germany; 2 Centre for Inflammation Research, Queen's Medical Research Institute, University of Edinburgh, Edinburgh, United Kingdom; 3 Institute for Inorganic Chemistry and Center for Nanointegration Duisburg-Essen (CeNIDE), University of Duisburg-Essen, Duisburg, Germany; 4 Institute of Infection, Immunity and Inflammation, University of Glasgow, Glasgow, United Kingdom; 5 Institute of Veterinary Pathology, Freie Universitaet Berlin, Berlin, Germany; New York University, UNITED STATES

## Abstract

Inflammatory bowel diseases (IBD) are chronic inflammatory disorders of the gastrointestinal tract, strongly associated with an increased risk of colorectal cancer development. Parasitic infections caused by helminths have been shown to modulate the host’s immune response by releasing immunomodulatory molecules and inducing regulatory T cells (Tregs). This immunosuppressive state provoked in the host has been considered as a novel and promising approach to treat IBD patients and alleviate acute intestinal inflammation. On the contrary, specific parasite infections are well known to be directly linked to carcinogenesis. Whether a helminth infection interferes with the development of colitis-associated colon cancer (CAC) is not yet known. In the present study, we demonstrate that the treatment of mice with the intestinal helminth *Heligmosomoides polygyrus* at the onset of tumor progression in a mouse model of CAC does not alter tumor growth and distribution. In contrast, *H*. *polygyrus* infection in the early inflammatory phase of CAC strengthens the inflammatory response and significantly boosts tumor development. Here, *H*. *polygyrus* infection was accompanied by long-lasting alterations in the colonic immune cell compartment, with reduced frequencies of colonic CD8^+^ effector T cells. Moreover, *H*. *polygyrus* infection in the course of dextran sulfate sodium (DSS) mediated colitis significantly exacerbates intestinal inflammation by amplifying the release of colonic IL-6 and CXCL1. Thus, our findings indicate that the therapeutic application of helminths during CAC might have tumor-promoting effects and therefore should be well-considered.

## Author summary

Evidence from epidemiological studies indicates an inverse correlation between the incidence of certain immune-mediated diseases, including inflammatory bowel diseases, and exposure to helminths. As a consequence, helminth parasites were tested for treating IBD patients, resulting in clinical amelioration of the disease due to the induction of an immunosuppressive microenvironment. However, some infection–related cancers can be attributed to helminth infection, probably due to the generation of a microenvironment that might be conductive to the initiation and development of cancer. In the present study, we aimed to unravel the apparently controversial function of helminths in a mouse model of colitis-associated colon cancer. We show that helminth infection in the onset of colitis and colitis-associated colon cancer does not ameliorate colonic inflammation but activates intestinal immune cells that further facilitate tumor development. Therefore, a better understanding of mechanisms by which helminths modulate host immune responses in the gut should be defined precisely before application of helminths in autoimmune diseases like IBD.

## Introduction

A close relationship between inflammation and tumor development has been described for numerous human cancers over the last years [1]. In terms of inflammatory bowel diseases (IBD) such as ulcerative colitis (UC), it has been clearly demonstrated that patients are predisposed to develop colorectal cancer [2]. The pathology of IBD is thought to be caused by either genetic susceptibility, environmental influences, infectious microbes, or dysregulated intestinal immune responses. Due to one or more of these factors, the tolerance towards dietary antigens and the commensal microbiota in the gastrointestinal tract are perturbed. Excessive inflammation is initiated by innate immune cells, but further pathology is driven by a prevalent activation of T helper cells (Th) 1, Th2 or Th17 cells [3]. While studying mouse models of intestinal inflammation, regulatory T cells (Tregs) were shown to play a critical role in maintaining mucosal homoeostasis. As they exert a variety of suppressive functions Tregs are able to prevent aberrant activation of intestinal immune responses [4]. In the murine model of colitis-associated colon cancer (CAC), CD4^+^ Foxp3^+^ Tregs are crucial for the control of the inflammatory process. However, during tumor progression, highly activated Tregs accumulate within colonic tumors and suppress CD8^+^ T cell antitumor responses effectively [5].

Since the etiology of IBD is still unknown and causative therapies aren’t available, the patients`exposure to helminths appeared to be a novel and promising approach in the treatment of colitis. During helminth infection, pronounced Th2 immune responses as well as an activation of B cells, basophils, mast cells, dendritic cells, and eosinophils are evoked in the host to control and expel the parasites. By the expansion of regulatory cells such as alternatively activated macrophages, CD4^+^ and CD8^+^ Tregs or regulatory B cells, and the consequent induction of anti-inflammatory cytokines, e.g. IL-10 or TGF-β, helminths constitute immunoregulatory conditions to ensure their survival [6]. This immunomodulatory state was suggested to limit intestinal inflammation in IBD. However, utilization of helminths in different human studies and animal experiments of colitis highlighted controversial results. First results of clinical trials of *Trichuris suis* ova (TSO) therapy in UC and Crohn`s disease patients showed a reduction of the disease activity index [7, 8]. In retrospect, evaluation of completed studies did not illustrate any significant beneficial effect of oral TSO application on clinical response or remission in Crohn’s disease or UC patients (ClinicalTrials.gov Identifier: NCT01433471, NCT01576471, NCT01953354). Therefore, all clinical trials were either terminated or completed without publication of final results. In contrast, murine models of *H*. *polygyrus* infection during DSS [9] or T cell transfer colitis [10] as well as the application of *Hymenolepis diminuta* in dinitrobenzene sulfonic acid induced colitis [11] illustrated an amelioration of the intestinal inflammation. In almost all studies, efficacy of treatment was accompanied by an expansion of Foxp3^+^ Tregs or a production of IL-10. Nevertheless, adverse effects of helminth application were also shown in different mouse models. Disease severity was enhanced when mice were infected with *H*. *diminuta* in oxazolone-induced colitis [12, 13], or after *Trichuris muris* infection of genetic susceptible mice which develop colitis spontaneously [14]. Moreover, *H*. *polygyrus* infection in a mouse model of bacterial colitis was shown to exacerbate intestinal inflammation by inducing alterations of the colonic microbiota [15].

Regardless of IBD, infections are known to account for many malignancies, including cancer, as they can induce chronic inflammation, genomic instability, inhibition of apoptosis or tumor suppressors, and modulation of cell proliferation, angiogenesis or glucose metabolism. With respect to helminths, data indicated that at least blood and liver flukes can be responsible for carcinogenesis. *Schistosoma haematobium* infections could be clearly linked to bladder cancer, and *Chlonorchis sinensis* or *Opisthorchis viverrini* infections have been shown to cause cholangiocarcinoma and bile duct cancer [16, 17]. Mechanisms that facilitate carcinogenesis in *O*. *viverrini* infections are the production of reactive oxygen intermediates which induce chronic inflammation and the release of proteins which promote cell proliferation and interfere with DNA repair and apoptosis pathways [18]. Whether an association between helminths other than flukes or schistosomes and cancer exists, remains unclear. For intestinal nematodes, like *T*. *suis*, which have already been applied to IBD patients, not much is known about their potential to exert long-term effects in the host.

Given that helminths potently suppress inflammation through versatile immunoregulatory mechanisms but simultaneously may facilitate carcinogenesis, we aimed to unravel the apparently controversial function of helminth infection in a mouse model of colitis-associated colon cancer. Thereby, we focused on the distribution and phenotype of Foxp3^+^ Tregs and CD8^+^ T cells which play an important role in tumor immunity.

## Results

### Helminth infection affects the frequency of CD4^+^ Foxp3^+^ Tregs in the colon

**Fig 1:**
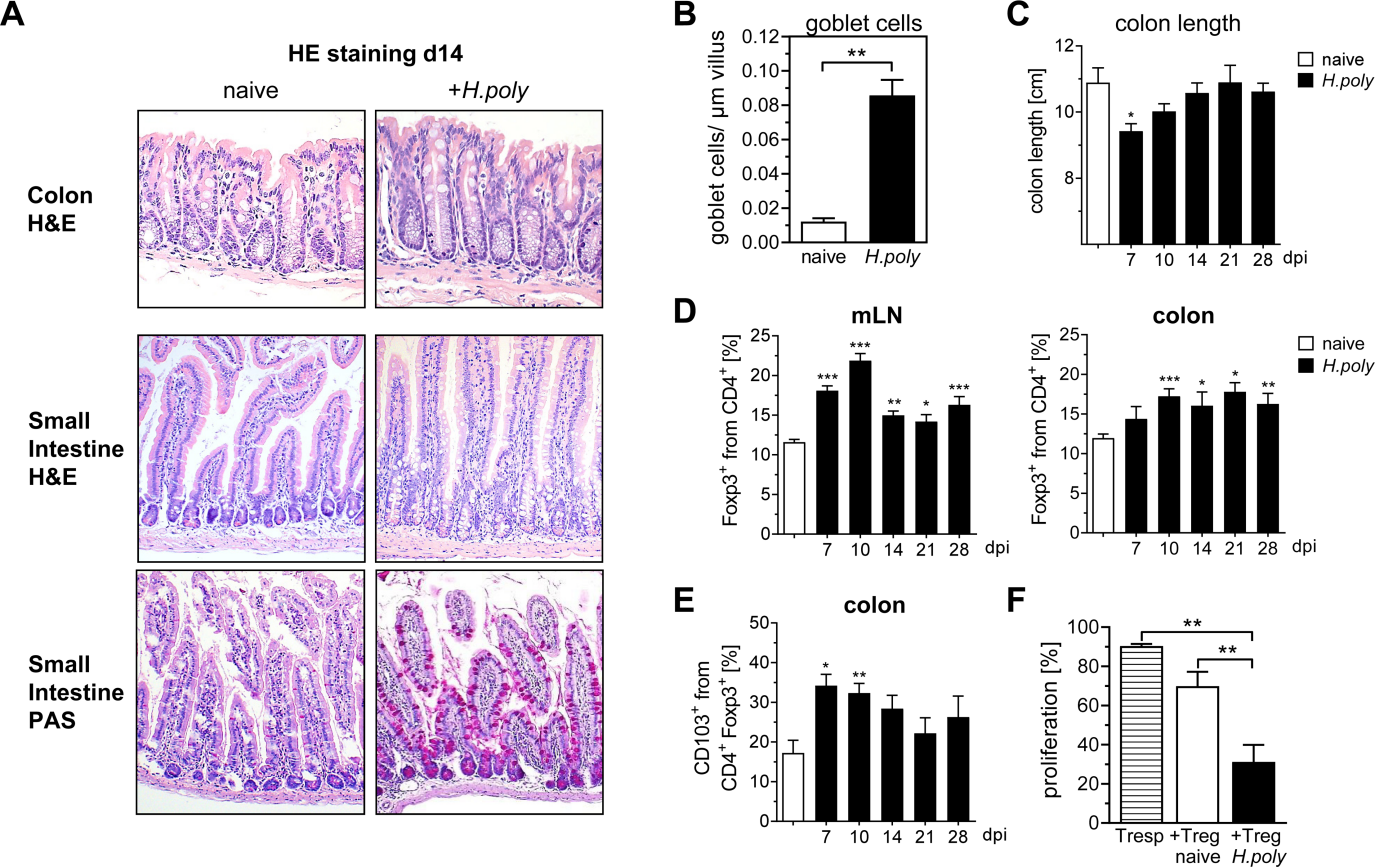
*H*. *polygyrus* infection leads to the expansion of regulatory T cells in the colon. BALB/c mice were infected with 200 stage-three larvae (L3) *H*. *polygyrus* by oral gavage, and at indicated time points post infection mice were sacrificed. (A) At day 14, representative tissue sections of the colon and the small intestine from naïve mice and *H*. *polygyrus* (*H*.*poly*) infected mice were fixed and stained with haematoxylin and eosin (H&E) or periodic acid Schiff (PAS) to show pathologic changes. Images show magnification at x200. (B) Goblet cells in PAS stained sections were counted and referred to villi length. Bars represent the mean±SEM of data from one experiment (naïve, n = 2; naïve+*H*.*poly*, n = 3). Statistical significance was calculated using unpaired t test (**, p≤ 0.01). (C) At indicated days post infection (dpi), colon from naïve mice and *H*. *polygyrus* infected mice were prepared and colon length was measured. Bars represent the mean±SEM of data from 2 independent experiments (naïve, n = 10; naïve+*H*.*poly*, n = 5). (D, E) At indicated dpi, mLNs and LPLs of naïve mice and *H*. *polygyrus* infected mice were isolated and stained for the expression of CD4, CD103 and intracellular Foxp3. Bars represent the mean±SEM of data from 3 independent experiments (naïve, n = 14; naïve+*H*.*poly*, n = 8). (F) To determine the suppressive capacity of Treg *in vitro*, Foxp3/eGFP mice were infected with 200 L3 *H*. *polygyrus*, and at day 10 post infection, CD4^+^eGFP^+^ (Foxp3^+^) T cells (Treg) from mLN of infected or naive mice were sorted. Tregs were co-cultured at a ratio of 1:1 with eFluor-labeled CD4^+^ responder T cells (Tresp) and with antigen-presenting cells in the presence of a-CD3. Proliferation of Tresp was measured by loss of eFluor dye. Bar diagram represents the proliferation as mean±SEM of 2 independent experiments (naïve, n = 7; *H*.*poly*, n = 7). Statistical significance was calculated using one-way ANOVA followed by Tukey's Multiple Comparison Test (*, p≤ 0.05; **, p≤ 0.01; ***, p≤ 0.001).

Intestinal infections with the parasitic nematode *H*. *polygyrus* are known to expand CD4^+^ Foxp3^+^ Tregs [19] and lead to chronic inflammation in susceptible mouse strains; two features that are considered to promote carcinogenesis in colitis-associated colon cancer [5]. Before we applied the intestinal nematode *H*. *polygyrus* in the AOM/DSS colon cancer mouse model, we tested whether helminth infection alone might modulate the host immune response distal to the site of infection, particularly in the colon. Fourteen days after infection of BALB/c mice with 200 third-stage larvae of *H*. *polygyrus*, we did not observe any mucosal damage but a slight induction of smooth muscle thickening in the colon (Fig 1A). This was in line with a minimal reduction of the colon length between 7 and 14 days post infection (Fig 1C). As expected, in the small intestine, where *H*. *polygyrus* larvae encyst, molt and reemerge as adult worms to the gut lumen, severe goblet cell hyperplasia was detected by day 14 post infection (Fig 1A and 1B). Intestinal helminth infections are considered to modulate CD4^+^ T cell subsets [20]. Thus, we further analyzed the T cell compartment of the mesenteric lymph nodes (mLNs) and colonic lamina propria (LP) at different time points after *H*. *polygyrus* infection. Besides a considerable expansion of CD4^+^ Foxp3^+^ Tregs in the mLN of infected mice, most prominent by day 10 post infection, we also detected increased frequencies of Tregs in the LP during the course of infection (Fig 1D). To explore the phenotype of these cells, we determined the expression of the integrin αE (CD103) on CD4^+^ Foxp3^+^ Tregs from naïve mice and mice infected with *H*. *polygyrus* as CD103^+^ Tregs were shown to represent an effector/memory phenotype [21]. The CD103 expression was significantly elevated on Tregs isolated from the colon already 7 days post infection and showed the highest increase at day 10 (Fig 1E). To determine whether this activated phenotype is correlated with a higher functional activity, we isolated CD4^+^ Foxp3^+^ Tregs from mLN at day 10 post infection and cultured them with CD4^+^ responder T cells. Indeed, Tregs isolated from *H*. *polygyrus* infected mice were more potent in the suppression of responder T cell proliferation than CD4^+^ Foxp3^+^ Tregs from naïve animals (Fig 1F).

### *H*. *polygyrus* infection does not impair tumor growth in established colitis-associated colon cancer

**Fig 2:**
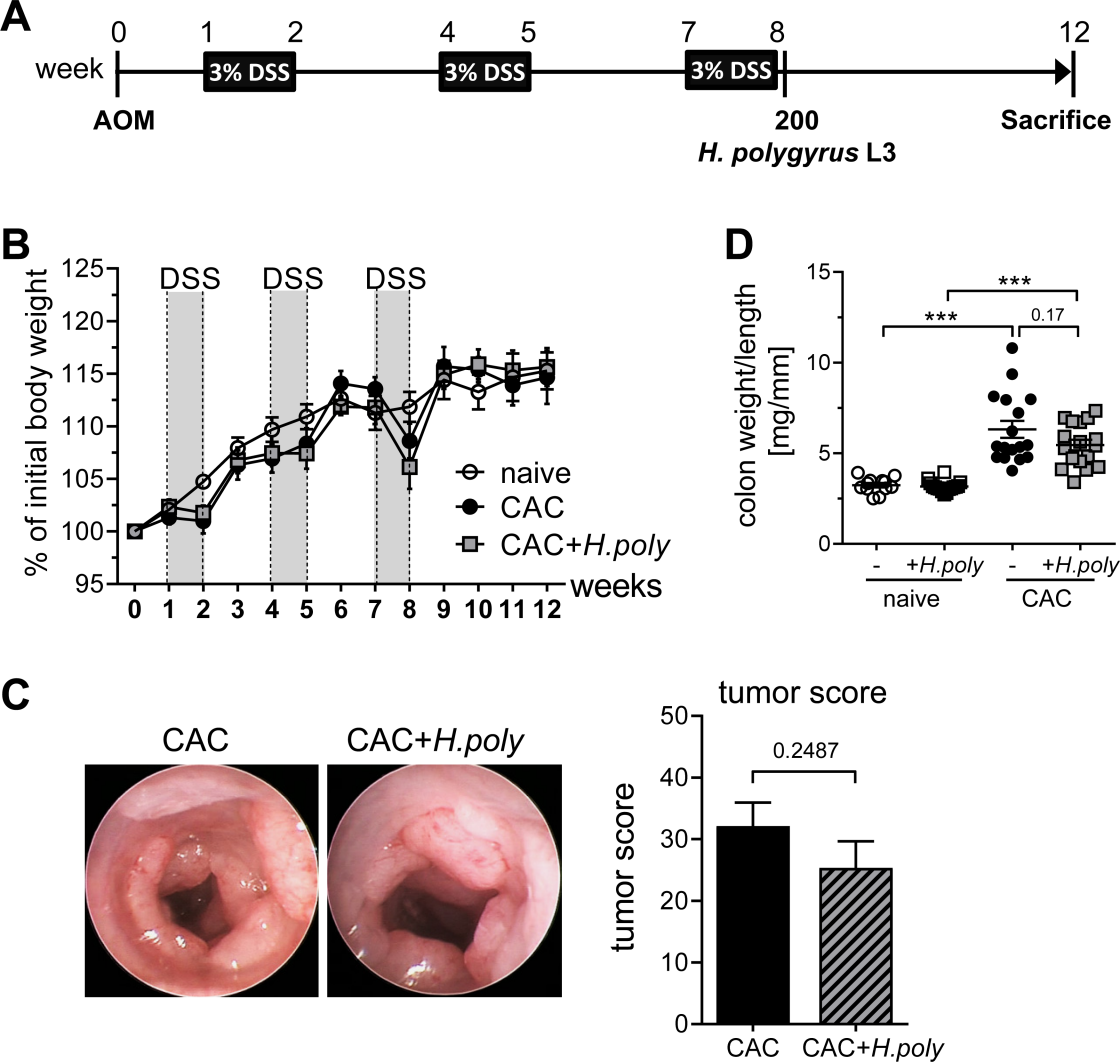
*H*. *polygyrus* infection in the late phase of colitis-associated colon cancer does not impact tumor growth. (A) Schematic time schedule of *H*. *polygyrus* (*H*.*poly*) infection and the induction of colitis-associated colon cancer (CAC) in BALB/c mice. After an intraperitoneal injection of the procarcinogen azoxymethane (AOM), 3 cycles of dextran sulfate sodium (DSS) were given via the drinking water. At week 8, mice were infected with 200 stage-three larvae (L3) *H*. *polygyrus* by oral gavage, and tumor development was analyzed at week 12. (B) Weight change of naïve mice (unfilled circles), AOM/DSS-treated mice (CAC, black circles) and AOM/DSS-treated *H*. *polygyrus* infected mice (CAC+*H*.*poly*, grey rectangles) relative to initial body weight during the course of the experiment. The graph shows data from 4 independent experiments (naïve, n = 12; CAC, n = 17; CAC+*H*.*poly*, n = 17). (C) Murine endoscopy was performed to obtain representative endoscopic images from the distal colon of CAC and CAC+*H*.*poly* mice and to determine tumor scores. Bars represent the mean±SEM of data from 4 experiments (CAC, n = 17; CAC+*H*.*poly*, n = 17). (D) Colon from naïve mice, *H*. *polygyrus* infected mice, CAC mice and CAC+*H*.*poly* mice were prepared to calculate colon weight to length ratios. Data from 4 independent experiments are shown (naïve, n = 12; naïve+*H*.*poly*, n = 16; CAC, n = 17; CAC+*H*.*poly*, n = 17). Statistical significance was calculated using one-way ANOVA followed by Tukey's Multiple Comparison Test (***, p≤ 0.001).

We previously demonstrated that CD4^+^ Foxp3^+^ Tregs elicit tumor-promoting functions during CAC in mice. More precisely, CD4^+^ Tregs were shown to be involved in the suppression of an effective CD8^+^ T cell-mediated antitumor response [5]. To investigate whether the alterations in Treg frequencies, which are provoked by *H*. *polygyrus* infection, might have an impact on the development of CAC, we infected BALB/c mice with 200 third-stage larvae of *H*. *polygyrus* at week 8 of the AOM/DSS regimen (Fig 2A). At this time point, the basis for adenocarcinomas had been created and tumor growth set in. As seen in Fig 2B, *H*. *polygyrus* infection at week 8 did not prejudice the weight course of the mice which is only dictated by the administration of DSS via the drinking water. When tumor development was monitored by endoscopy at week 12, no differences in the frequencies or sizes of adenocarcinomas as indicated by the tumor score were observed (Fig 2C). Due to the tumor growth, the colon weight to length ratio was increased in AOM/DSS treated mice. However, additional *H*. *polygyrus* infection had no further influence on the carcinogenesis (Fig 2D).

### Tumor development during colitis-associated colon cancer is boosted by early stage *H*. *polygyrus* infection

**Fig 3:**
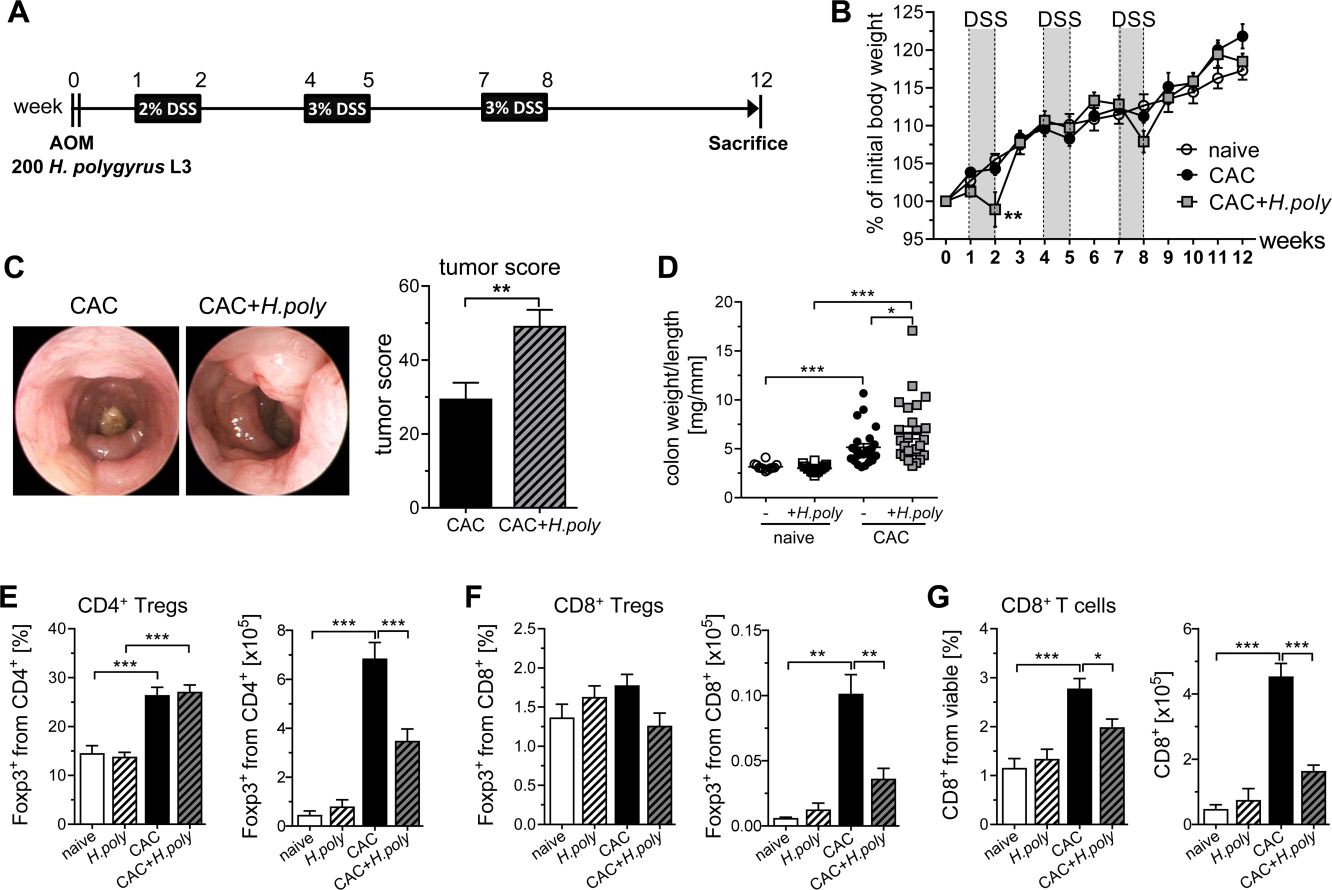
*H*. *polygyrus* infection in the early inflammatory phase of colitis-associated colon cancer increases tumor development. (A) Schematic time schedule of *H*. *polygyrus* (*H*.*poly*) infection and the induction of colitis-associated colon cancer (CAC) in BALB/c mice. One day after intraperitoneal injection of the procarcinogen azoxymethane (AOM), mice were infected with 200 stage-three larvae (L3) *H*. *polygyrus* by oral gavage. After 6 days, dextran sulfate sodium (DSS) was given via the drinking water. DSS administration was repeated twice, and mice were analyzed at week 12. (B) Weight change of naïve mice (unfilled circles), AOM/DSS-treated mice (CAC, black circles) and AOM/DSS-treated *H*. *polygyrus* infected mice (CAC+*H*.*poly*, grey rectangles) relative to initial body weight during the course of the experiment. The graph shows data from 5 independent experiments (naïve, n = 12; CAC, n = 24; CAC+*H*.*poly*, n = 25). Statistical significance was calculated using two-way ANOVA and Bonferroni posttests (**, p≤ 0.01). (C) Murine endoscopy was performed to obtain representative endoscopic images from the distal colon of CAC and CAC+*H*.*poly* mice and to determine tumor scores. Bars represent the mean±SEM of data from 5 experiments (CAC, n = 24; CAC+*H*.*poly*, n = 25). Statistical analyses were performed by unpaired t test (**, p≤ 0.01). (D) At week 12, Colon from naïve mice, *H*. *polygyrus* infected mice, CAC mice and CAC+*H*.*poly* mice were prepared to calculate colon weight to length ratios. LPLs from the colons were isolated and stained for the expression of CD4, CD8, and intracellular Foxp3. Frequencies of (E) CD4^+^Foxp3^+^ Tregs, (F) Foxp3^+^ CD8^+^ Tregs and (G) CD8^+^ T cells were determined by flow cytometry and absolute numbers were calculated. Graphs represent the mean±SEM of data from 5 experiments (naïve, n = 12; naïve+*H*.*poly*, n = 16; CAC, n = 24; CAC+*H*.*poly*, n = 25). Statistical significance was calculated using one-way ANOVA followed by Tukey's Multiple Comparison Test (*, p≤ 0.05; **, p≤ 0.01; ***, p≤ 0.001).

By increasing the number of T cells expressing Foxp3 in the colon, *H*. *polygyrus* infection was shown to protect mice in some models of experimental colitis [10, 22]. On the contrary, helminth infections themselves modulate their host´s immune system, which may favor tumor development. In order to examine the influence of *H*. *polygyrus* infection on DSS-induced inflammation during CAC we infected BALB/c mice with 200 third-stage larvae of *H*. *polygyrus* one day after AOM injection, 6 days before the first DSS administration, respectively (Fig 3A). Interestingly, we observed a significantly accelerated loss of body weight during the first DSS cycle in mice infected with *H*. *polygyrus*, indicating more severe intestinal inflammation (Fig 3B). Colonoscopy at week 12 revealed that mice infected with *H*. *polygyrus* before the first DSS administration exhibited a significantly higher tumor score compared to AOM/DSS treated mice (Fig 3C). Augmented tumor formation was accompanied by a significant gain of colon weight to length ratios in *H*. *polygyrus* infected CAC mice compared to non-infected CAC mice (Fig 3D). Due to the increased frequencies of Tregs in the colonic lamina propia during the course of infection, we speculated whether alterations in the colonic cellular composition might be responsible for the enhanced tumor development after *H*. *polygyrus* infection. As expected, mice suffering from CAC showed a strong accumulation of Foxp3^+^ Tregs in the colon (Fig 3E and 3F). However, no additive effect was observed when mice were infected with *H*. *polygyrus*. In contrast, absolute numbers of Foxp3^+^ Tregs was significantly decreased in the colon of *H*. *polygyrus* infected CAC mice compared to CAC mice (Fig 3E and 3F). This could be ascribed to the overall reduction of cells in the colon of *H*. *polygyrus* infected CAC mice as also the frequencies and absolute numbers of CD8^+^ effector T cells were significantly diminished in the colon of infected CAC mice, although the initial *H*. *polygyrus* infection was induced 12 weeks earlier (Fig 3G). In summary, these data show that *H*. *polygyrus* infection seems to have a long-lasting impact on the anti-tumor immune response, but the increase in the tumor score after *H*. *polygyrus* is not dependent on an enhanced Treg frequency.

### *H*. *polygyrus* infection enhances DSS-induced inflammation in the colon

**Fig 4:**
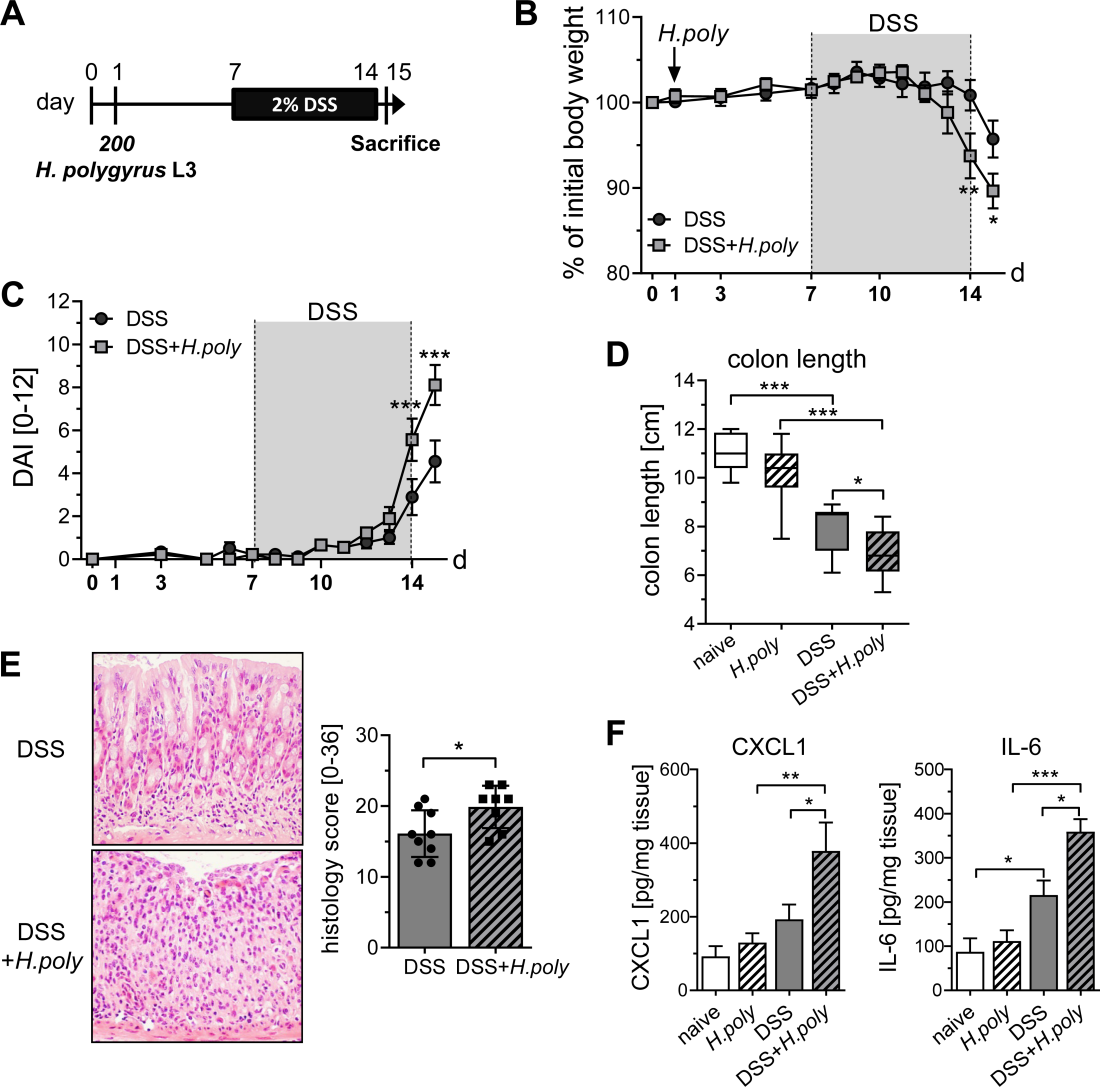
DSS-induced acute colonic inflammation is augmented by *H*. *polygyrus* infection. (A) Schematic time schedule of *H*. *polygyrus* (*H*.*poly*) infection and induction of dextran sulfate sodium colitis (DSS) in BALB/c mice. At day 1, mice were infected with 200 stage-three larvae (L3) *H*. *polygyrus* by oral gavage. Six days later, DSS was given via the drinking water for 7 days and mice were sacrificed on day 15. Weight change (B) of DSS-treated mice (DSS, black circles) and DSS-treated *H*. *polygyrus* infected mice (DSS+*H*.*poly*, grey rectangles) relative to initial body weight and disease activity index (C) during the course of the experiment. The graph shows data from 2–4 independent experiments (DSS, n = 9–15; DSS+*H*.*poly*, n = 9–15). Statistical significance was calculated using two-way ANOVA and Bonferroni posttests (*, p≤ 0.05; **, p≤ 0.01; ***, p≤ 0.01). (D) At day 15 colons were prepared and the length was measured. Box plots represent the median (horizontal lines), 10th to 90th percentile (extension of boxes), and range (error bars) of n = 9–15 mice per group. Statistical significance was calculated using one-way ANOVA followed by Tukey's Multiple Comparison Test (*, p≤ 0.05; ***, p≤ 0.001). (E) At day 15, representative tissue sections of colon samples from DSS mice and DSS+*H*. *poly* mice were fixed and stained with haematoxylin and eosin to show pathologic changes. Images show magnification at x200. Severity of colitis was assessed by scoring pathological changes. Bars show the mean ± SEM of the histological score from 2 experiments (DSS, n = 9; DSS+*H*.*poly*, n = 8). Statistical analyses were performed by unpaired t test (*, p≤ 0.05). (F) Biopsies from colon samples of naïve mice, *H*. *polygyrus* infected mice, DSS mice and DSS+*H*. *poly* mice were cultured *in vitro* for 6 hours in culture medium. Levels of IL-6 and CXCL1 in the supernatants were determined by Luminex. Bars show the mean ± SEM of cytokines per milligram tissue from 3 experiments (naïve, n = 10; naïve+*H*.*poly*, n = 12; DSS, n = 11; DSS+*H*.*poly*, n = 11). Statistical significance was calculated using one-way ANOVA followed by Tukey's Multiple Comparison Test (*, p≤ 0.05; **, p≤ 0.01; ***, p≤ 0.001).

Interestingly, we observed that mice subjected to AOM/DSS treatment and *H*. *polygyrus* infection before the first DSS administration, exhibited a stronger loss of body weight compared to AOM/DSS treated mice during the first DSS cycle (Fig 3B). This observation indicated a more severe intestinal inflammation in *H*. *polygyrus* infected CAC mice, which was shown to enhance tumor formation in this experimental mouse model [23, 24]. To elucidate the magnitude of inflammation, we infected BALB/c mice with 200 third-stage larvae of *H*. *polygyrus* 6 days before administration of 2% DSS in the drinking water (Fig 4A). In accordance with our hypothesis, *H*. *polygyrus* infected DSS treated mice started to loose body weight earlier and had significantly reduced body weights at day 14 when compared to DSS only treated mice (Fig 4B). The determination of the disease activity index, which consists of body weight loss, stool consistency and rectal bleeding, confirmed that *H*. *polygyrus* infected mice are significantly more sensitive to DSS-induced intestinal inflammation than non-infected mice (Fig 4C). Increased colonic inflammation in *H*. *polygyrus* infected DSS treated mice was associated with a significant reduction in colon length and enhanced pathology characterized by more severe loss of the entire crypt structure, surface epithelial cell erosion, goblet cell depletion and massive leukocyte infiltration compared to DSS only treated mice (Fig 4D and 4E). Well in line, the production of IL-6 and CXCL1 was significantly enhanced in the colon of infected DSS treated mice compared to non-infected DSS treated mice (Fig 4F). In contrast to the pathological changes in the colon, no signs of DSS-induced pathology were observed in the small intestine, but *H*. *polygyrus* mediated goblet cell hyperplasia was present (S1A and S1B Fig). Cytokine and chemokine production in intestinal explant cultures from the ileum confirmed that the DSS administration did not alter constitutive IL-6 or CXCL1 expression in the small intestine. However, *H*. *polygyrus* infected and DSS treated mice produced significantly lower levels of IL-6 and CXCL1 compared to DSS treated mice, suggesting an at least partly immunoregulatory environment in the small intestine (S1C Fig).

**Fig 5:**
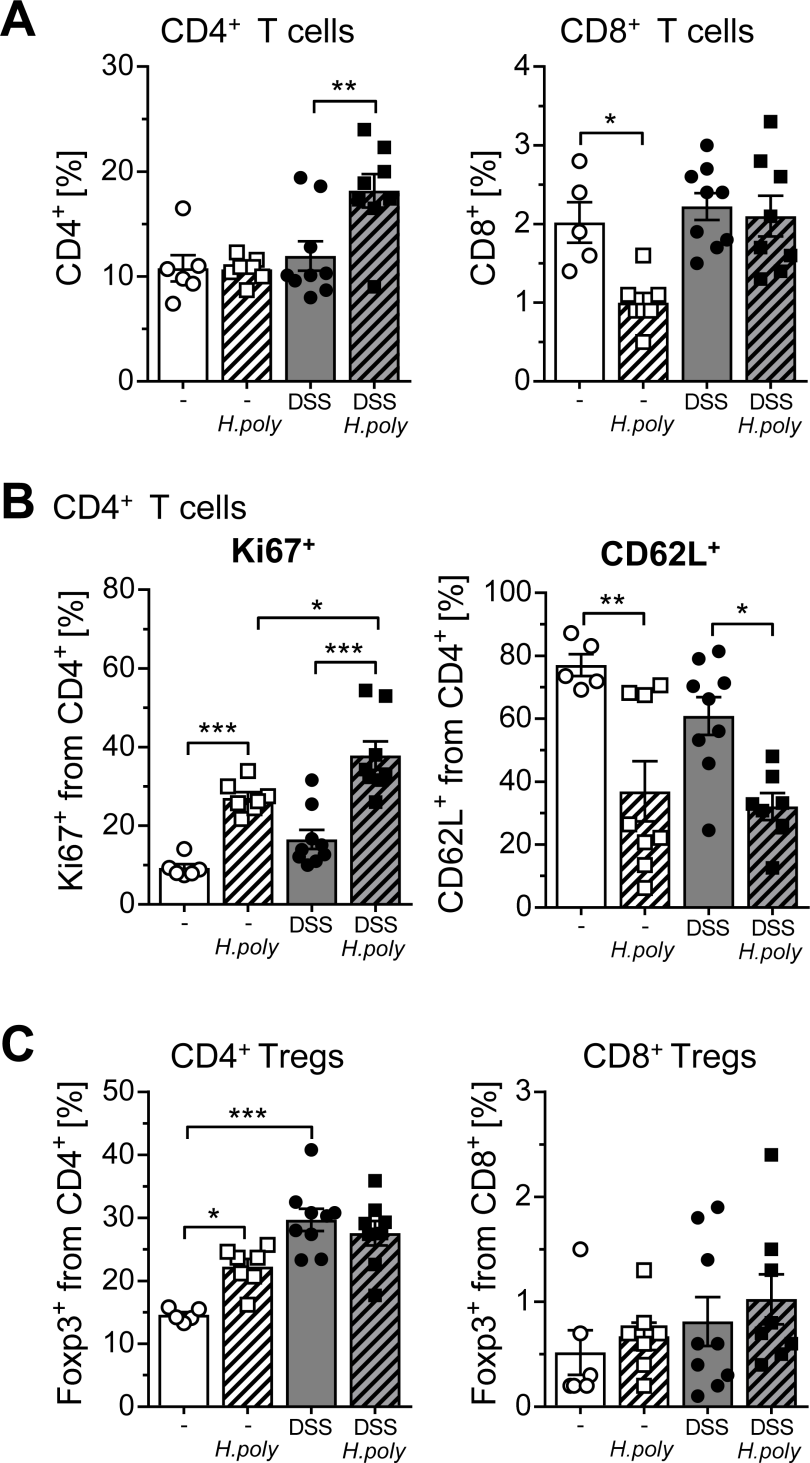
*H*. *polygyrus* infection activates colonic effector T cells during DSS-induced acute colitis. BALB/c mice were infected with *H*. *polygyrus* and subjected to acute DSS colitis 6 days later. At day 15, LPLs from the colons of naïve mice, *H*. *polygyrus* infected mice, DSS mice and DSS+*H*. *poly* mice were isolated and stained for the expression of CD4, CD8, CD62L, and intracellular Ki67 and Foxp3. Frequencies of (A) CD4^+^ or CD8^+^ T cells and (B) expression of CD62L and Ki67 were assessed among Foxp3^-^CD4^+^ T cells. (C) Frequencies of Foxp3^+^ among CD4^+^ T cells or CD8^+^ T cells were determined by flow cytometry. Bars represent the mean±SEM of data from 3 experiments (naïve, n = 6; naïve+*H*.*poly*, n = 7; DSS, n = 9; DSS+*H*.*poly*, n = 8). Statistical significance was calculated using one-way ANOVA followed by Tukey's Multiple Comparison Test (*, p≤ 0.05; **, p≤ 0.01; ***, p≤ 0.001).

Determining the cellular composition in the colonic lamina propria we detected the highest frequency of CD4^+^ T cells in *H*. *polygyrus* infected DSS treated mice (Fig 5A). The strong expression of the proliferation marker Ki67 and the downregulation of CD62L on CD4^+^ Foxp3^-^ T cells in the colon of *H*. *polygyrus* infected DSS treated mice reflected a significantly enhanced activation of CD4^+^ Foxp3^-^ T cells which was independent of DSS application (Fig 5B). Interestingly, the frequency of CD8^+^ T cells was significantly reduced in the colon of *H*. *polygyrus* infected mice but not in DSS treated infected mice (Fig 5A). Finally, the frequency of Foxp3^+^ Tregs was increased in the colon of *H*. *polygyrus* infected mice, which was further enhanced by DSS-treatment (Fig 5C). In summary, we conclude that *H*. *polygyrus* infection prior to DSS-induced colitis further enhances the inflammatory response in the colon based on enhanced IL-6 and CXCL1 expression and increased CD4^+^ T cell activation. As these results are unexpected and in part in contrast to the published literature we made use of two other mouse models for colonic inflammation to validate our results. We used the RAG2^-/-^ T cell transfer colitis model [25] and the VILLIN-HA T cell transfer model [26]. Based on our experimental set up, mice were infected with *H*. *polygyrus* before T cell transfer and the pathology in the colon was analyzed. Well in line with the results obtained in the DSS colitis model, we absolutely not observed any improvement in the disease outcome after *H*. *polygyrus* infection (S2A–S2E Fig). In contrast, *H*. *polygyrus* pre-infection in the RAG2^-/-^ T cell transfer colitis model seems to induce IL-6 and CXCL1 in the colon in the same manner as in the DSS model (S2C Fig).

### *In vivo* neutralization of IL-6 and CXCL1 reduces severity of DSS-induced inflammation in *H*. *polygyrus* infected mice

**Fig 6:**
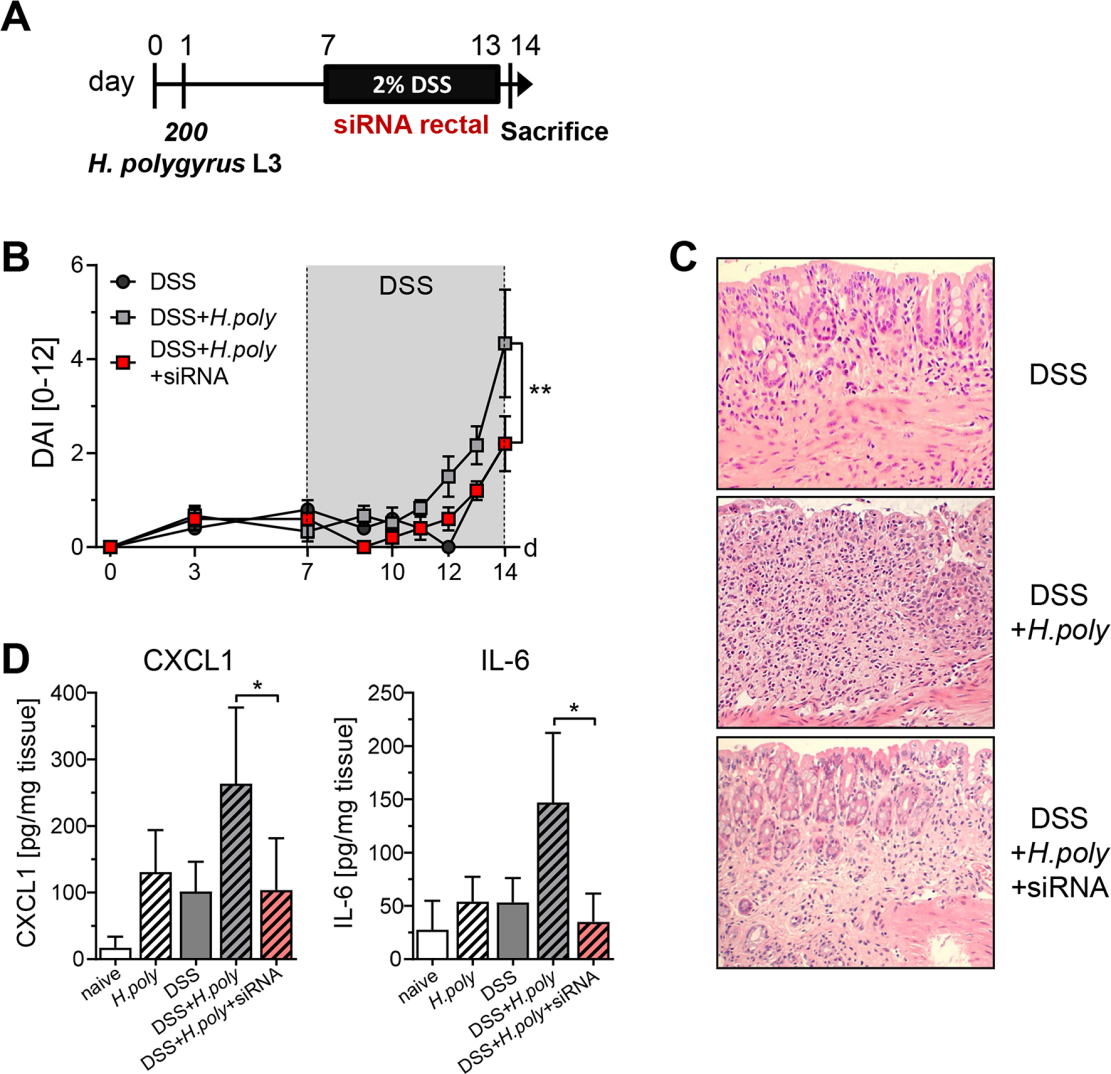
Gene silencing of IL-6 and CXCL1 ameliorates colonic inflammation in *H*. *polygyrus* infected DSS treated animals. (A) Schematic time schedule of *H*. *polygyrus* (*H*.*poly*) infection and siRNA treatment during the induction of dextran sulfate sodium colitis (DSS) in BALB/c mice. At day 1, mice were infected with 200 stage-three larvae (L3) *H*. *polygyrus* by oral gavage. Six days later, DSS was given via the drinking water for 7 days and mice were sacrificed on day 14. Eight μg siRNA-loaded CaP/PLGA nanoparticles directed against IL-6 and CXCL1 were applied intrarectally daily during DSS treatment until day 13. (B) Disease activity index (DAI) of DSS-treated mice (DSS, black circles), DSS-treated *H*. *polygyrus* infected mice (DSS+*H*.*poly*, grey rectangles) and siRNA+DSS-treated *H*. *polygyrus* infected mice (DSS+*H*.*poly*+siRNA, red rectangles) during the course of the experiment. The graph shows data from 1 experiment. Statistical significance was calculated using two-way ANOVA and Bonferroni posttests (**, p≤ 0.01). (C) Representative tissue sections of colon samples from DSS, DSS+*H*. *poly* and DSS+*H*.poly+siRNA mice were fixed and stained with haematoxylin and eosin to show pathologic changes. Images show magnification at x200. (D) At day 14 colons were prepared and biopsies from colon samples were cultured *in vitro* for 6 hours in culture medium. Levels of IL-6 and CXCL1 in the supernatants were determined by Luminex. Bars show the mean ± SEM of cytokines per milligram tissue from 1 experiment (DSS, n = 5; DSS+*H*.*poly*, n = 6; DSS+*H*.*poly*+siRNA, n = 5). Statistical significance was calculated using Mann Whitney test (*, p≤ 0.05).

Pro-inflammatory cytokines like IL-6 have important implications in driving intestinal inflammation [27]. Moreover, IL-6 is a critical tumor promoter during early tumorigenesis in the CAC mouse model [28]. To analyze if the helminth mediated increase in IL-6 and CXCL1 promoted the increased disease severity of DSS-induced colitis, we infected BALB/c mice with 200 third-stage larvae of *H*. *polygyrus* 6 days before DSS administration. In addition, mice were rectally treated with siRNA-loaded nanoparticles directed against CXCL1 and IL-6 during DSS application (Fig 6A) and the disease activity index was monitored. Importantly, inhibition of *H*. *polygyrus* mediated IL-6 and CXCL1 expression by siRNA significantly reduced the disease activity index to the level of DSS only treated mice (Fig 6B). Less severe inflammation was confirmed in colonic tissue sections as infected mice treated with siRNA-loaded nanoparticles showed a more preserved colonic structure compared to non- siRNA-treated mice (Fig 6C). Efficient gene silencing of IL-6 and CXCL1 was determined in the supernatant of colon explant cultures (Fig 6D). Our results clearly underline that *H*. *polygyrus* infection mediated colonic IL-6 and CXCL1 release facilitates aggravation of DSS-induced colitis.

### Intestinal inflammation and colitis-associated colon cancer impairs the anti-helminth response

**Fig 7:**
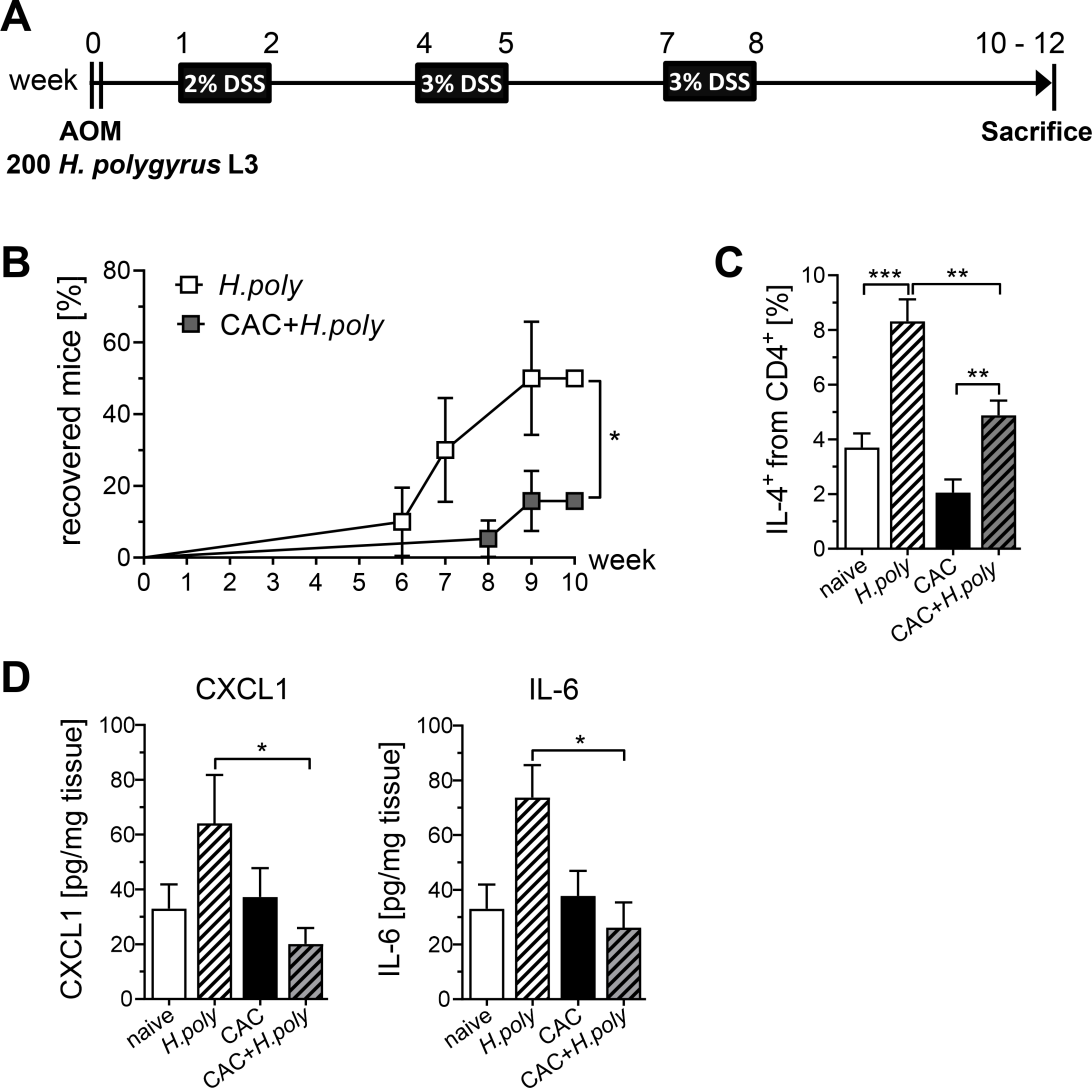
Colitis-associated colon cancer induction impacts the course of *H*. *polygyrus* infection. (A) Schematic time schedule of *H*. *polygyrus* (*H*.*poly*) infection and CAC induction in BALB/c mice. One day after i.p. injection of azoxymethane (AOM), mice were infected with 200 stage-three larvae (L3) *H*. *polygyrus*. After 6 days, dextran sulfate sodium (DSS) was given via the drinking water. DSS administration was repeated twice, and mice were analyzed at week 10 to 12. (B) Fecal egg counts were measured weekly during the course of the experiment until mice were sacrificed at week 10. Mice were graded as recovered when no eggs could be counted in their feces. The graph shows the percentage of recovered mice from 3 experiments with n = 10 *H*. *polygyrus* infected mice (open rectangles) and n = 19 CAC+*H*. *polygyrus* infected mice (grey rectangles) as survival curve. Statistical significance was calculated using log-rank test (*, p≤0.05). (C) At week 12, LPLs from the colons were isolated and stained for the expression of CD4 and intracellular IL-4. Frequencies of IL-4^+^ CD4^+^ T cells were determined by flow cytometry. (D) Colon biopsies were incubated *in vitro* for 6 hours in culture medium. Levels of IL-6 and CXCL1 in the supernatants were determined by Luminex and cytokines per milligram tissue were calculated. Bars represent the mean±SEM of data from 2–3 experiments (naïve, n = 4; naïve+*H*.*poly*, n = 6; CAC, n = 10–15; CAC+*H*.*poly*, n = 10–15). Statistical significance was calculated using one-way ANOVA followed by Dunn’s or Tukey's Multiple Comparison Test (*, p≤ 0.05; **, p≤ 0.01; ***, p≤ 0.001).

Our results definitely indicated that *H*. *polygyrus* infection facilitates intestinal inflammation in the AOM/DSS model. However, it is unclear whether intestinal inflammation or the immunosuppressive environment during colon cancer impacts the anti-helminth immune response and thereby alters the course of infection. In order to clarify this issue, we measured the fecal egg counts weekly in *H*. *polygyrus* infected mice and in mice treated with the AOM/DSS regime after *H*. *polygyrus* infection over the course of the experiment (Fig 7A). Interestingly, helminths persisted longer in mice suffering from intestinal inflammation and colon cancer than in naïve mice. At week 10, only 15% of CAC mice had recovered from infection while 50% of naïve mice showed expulsion of the worms (Fig 7B). Subsequent cytokine and chemokine analysis revealed that *H*. *polygyrus* infection in naïve mice elicited a Th2 response in the colon, indicated by a strong IL-4 release, important for the clearance of the parasites. In line with delayed clearance, *H*. *polygyrus* infected mice suffering from colon cancer produced significantly less IL-4 in response to the parasites (Fig 7C). Of note, *H*. *polygyrus* infection promoted a long term up-regulation of IL-6 and CXCL1 expression in naïve mice which was still detectable 10 weeks after initial infection. However, the immunosuppressive environment of tumors in AOM/DSS treated mice completely abolished the expression IL-6 and CXCL1 (Fig 7D), suggesting that colon cancer also affects the helminth-specific immune response.

Taken together, we show that *H*. *polygyrus* infection can amplify intestinal inflammation and induces long-lasting alterations in the colonic immune cell compartment, thereby increasing the risk to develop colitis-associated colon cancer.

## Discussion

Carcinogenesis in colitis-associated colon cancer is clearly driven by inflammation [1]. Therefore, controlling the inflammatory immune response is of major interest in IBD patients. Great effort has been done to find alternative drugs which do not solely alleviate the symptoms but interfere with the innate or adaptive immune response to restore intestinal homeostasis. Parasitic helminth infections became a subject of murine and human studies in IBD as they release immunomodulatory molecules that might reduce intestinal inflammation. However, systematic parasite infections in patients should be well-considered as they have been shown causative for different types of cancers [17]. In developing countries, autoimmune diseases such as IBD may be rare, but soil-transmitted helminth infections affect approximately one-third of the population [29], and 22.9% of cancers diagnosed were attributable to infections [30]. With regard to parasitic intestinal infections elicited by nematodes, almost nothing is known about their ability to cause cancer.

In the present study, we addressed the impact of *H*. *polygyrus* infection in the context of colitis-associated colon cancer (CAC). Importantly, we could show that, dependent on the time point of helminth infection, tumor development was strongly promoted. In contrast to our hypothesis, this effect was not due to a long-term increase in the frequency of immunosuppressive Foxp3^+^ Tregs in the colon, which are induced during *H*. *polygyrus* infection. We identified that infection with *H*. *polygyrus* in the early phase of CAC, before DSS-induced inflammation, accelerates the inflammatory immune response in the colon, leading to severe pathology, which were shown to enhance tumor development in this model [23, 24].

This is in contrast to other studies, which describe a protective role of helminths in different mouse models of IBD [31–33]. To validate our own data, we expanded our studies from the chemical induced IBD model to two T cells mediated IBD mouse model. Most important, in none of our model systems the pre-infection with *H*. *polygyrus* resulted in a protective effect and reduced inflammation in the colon. Therefore, the discrepancy to former studies must be dependent on other reasons e.g. in the way of application, the time point of infection or the nematodes that were used. In detail, attenuation of DSS-mediated inflammation was found when extracts or excretory/secretory products of helminths were applied [34, 35]. Furthermore, the *H*. *polygyrus*-mediated alleviation of colitis in the RAG IL-10^−/−^ T cell transfer colitis was observed, when *H*. *polygyrus* infected mice were dewormed before T cell transfer colitis [31]. These data strongly suggest that helminth products can be considered anti-inflammatory, rather than the helminths itself. Only one study described a protection against DSS colitis after infection with *H*. *polygyrus* larvae [9]. However, the authors infected the mice in the course of DSS treatment and analyzed the clinical outcome 5 days post infection. In contrast, we determined the clinical outcome at later time points, as we measured the inflammatory score two weeks after infection, and in the T cell-mediated colitis models we looked 3 weeks and 9 weeks, respectively, after initial infection. Therefore, the long-term effect of *H*. *polygyrus* infection seems to be different from the short-term effect, but must be strongly considered in this context. In line with our results, experiments with *H*. *diminuta* infection in oxazolone colitis demonstrated also an enhanced inflammatory response [12, 13]. Moreover, in a very recent study analyzing the impact of *H*. *polygyrus* pre-infection on bacterial colitis the authors showed that the infection with *H*. *polygyrus* led to significant changes in the microbiota composition which resulted in exacerbation of intestinal inflammation [15]. Whether alterations in the microbiota account for the increase in pro-inflammatory mediators in the colon and the boost of colitis severity needs further investigation.

During DSS-induced colitis, neither the presence of functional T cells, B cells and NK cells, nor commensal bacteria are crucial for the initiation of inflammatory processes [36, 37]. Nevertheless, in specific-pathogen-free (SPF) mice the disruption of the epithelial barrier by DSS leads to the activation of innate immune cells that sense commensal bacteria and initiate inflammation. CD4^+^ T cells are known to accumulate in the intestine during IBD [3, 38], and during DSS colitis, T cells that are specific against oral antigens develop [39]. In the current study, we attempted to identify the influence of *H*. *polygyrus* infection on the immunological response in DSS-induced colitis. Interestingly, we could not observed difference in the frequency of Tregs between *H*. *polygyrus* infected and non-infected mice suffering from inflammation, but the activation and proliferation of colonic CD4^+^ effector T cells was only observed when DSS treated mice were infected with *H*. *polygyrus*. DSS colitis itself did not expand or activate CD4^+^ T cells to a significant extent. In addition, high level of pro-inflammatory IL-6 and CXCL1 were produced in the colon of *H*. *polygyrus* DSS treated mice. In IBD patients IL-6 serves as a prognostic marker as increased serum levels correlate with enhanced clinical disease activity. In this context, IL-6 acts as a pro-inflammatory mediator that contributes to enhanced T cell survival and resistance to apoptosis by inducing STAT3 activation [40, 41]. Furthermore, IL-6 facilitates the expansion of Th17 cells which are IL-6 producers themselves and may have a pathogenic role in intestinal inflammation [42]. CXCL1 is highly expressed in the colonic mucosa of IBD patients and its pathophysiological role could be shown in mice lacking its receptor CXCR2. These mice displayed amelioration of DSS colitis accompanied by reduced infiltration of leucocytes to the colon [43]. Well in line, we show that elevated levels of IL-6 and CXCL1 are causative to *H*. *polygyrus* induced amplification of intestinal inflammation as the inhibition of IL-6 and CXCL1 expression by siRNA functionalized nanoparticles significantly reduced the disease activity in *H*. *polygyrus* infected DSS treated mice. Exacerbated inflammation is well known to promote tumor formation. In this context, proliferation of tumor-initiating cells is enhanced by IL-6 while normal and premalignant intestinal epithelial cells are protected from apoptosis [28]. Furthermore, the chemokine CXCL1 was demonstrated to promote angiogenesis in colorectal cancer [44].

Our data demonstrate that an augmented inflammation can be linked to a considerable increase of tumor growth. We have already shown that in the murine CAC model CD8^+^ cytotoxic T cells are crucial for the adaptive anti-tumor immune responses, while Foxp3^+^ Tregs facilitate immunosuppression [5]. Interestingly, *H*. *polygyrus* infection during CAC development induced a long-lasting reduction of CD8^+^ T cell frequencies in the colon. To our knowledge, our study is the first to describe an association of *H*. *polygyrus* infection and a prolonged CD8^+^ T cell reduction in the context of carcinogenesis. However, our findings are well in line with observations from murine and human helminth co-infection models. Helminth infection in patients suffering from tuberculosis or application of *T*. *suis* eggs in multiple sclerosis patients led to a decrease of CD8^+^ T cells in the peripheral blood [45, 46]. In mice infected with *Ascaris sp*. and Vaccinia virus, where CD8^+^ T cell responses are critical for host protection, decreased frequencies of CD8^+^ T cells and an increase of mortality were detected [47]. For *H*. *polygyrus* infection, it could be shown that CD8^+^ T-cell responses against *T*. *gondii* were suppressed and could not be restored [48]. When CD8^+^ effector T cells were adoptively transferred in *H*. *polygyrus* infected mice, their numbers and memory development were negatively impacted [49]. How precisely helminth infections modulate CD8^+^ T cells responses, needs to be further investigated.

*H*. *polygyrus* infection induces strong Th2 immune responses [50]. An increase of IL-4 producing CD4^+^ T cells could be detected in the colon several weeks after *H*. *polygyrus* infection. Remarkably, establishment of CAC significantly reduced the IL-4 response of CD4^+^ T cells after helminth infection which was accompanied by a prolonged survival of the worms. An altered cytokine milieu during intestinal inflammation might therefore impact immunogenicity of the helminths. This could be confirmed in a study where DSS administration was shown to enhance the adaptation and establishment of *H*. *polygyrus* indicated by an elevated egg production, increased worm length and altered distribution of larvae in the small intestine [51]. Cytokines measured in colon explant cultures from long-term *H*. *polygyrus* only infected mice showed a trend towards elevated IL-6 and CXCL1 levels (Fig 7D). In contrast, CAC induction after *H*. *polygyrus* infection significantly reduced production of IL-6 and CXCL1 in the colon compared to naïve infected mice. These results seem to be surprising as one would expect that tumor progression in infected CAC mice is connected to higher levels of IL-6 and CXCL1. However, it is possible that these pro-inflammatory mediators promote inflammation at early stages of CAC development and this is sufficient to enhance carcinogenesis in a long-term perspective.

In conclusion, we have shown that *H*. *polygyrus* infection in the context of DSS-induced colitis and CAC did not ameliorate colonic inflammation but activates intestinal immunity that facilitates tumor development. The data suggest that *H*. *polygyrus* infection not only interferes with CD4^+^ T cell responses but directly impacts on CD8^+^ effector T cells. Thus, mechanisms of helminths immune modulation should be defined precisely before being applied in autoimmune diseases like IBD.

## Materials and methods

### Ethics statement

All animal experiments were performed in accordance with institutional, state, and federal guidelines (approved by the Landesamt fuer Natur, Umwelt und Verbraucherschutz North Rhine-Westphalia, Germany; reference number: 84–02.04.2014.A243 and 87–51.04.2010.A163).

### Mice

Six to eight week old female BALB/c mice were purchased from Envigo (Rossdorf, Germany). Female C.Cg-Foxp3^tm2Tch^/J (termed Foxp3/eGFP) mice and CBy.PL(B6)-Thy1^a^/ScrJ (termed Thy1.1) mice (The Jackson Laboratory, Bar Harbor, ME) were bred in-house and used at an age of six to ten weeks. Animals were housed under specific pathogen-free conditions in the Laboratory Animal Facility of the University Hospital Essen.

### Induction of colitis-associated colon cancer and murine colonoscopy

To induce CAC, mice were injected intraperitoneally with a single dose of azoxymethane (AOM, 12.5 mg/kg of body weight; Sigma-Aldrich, Munich, Germany), followed by 3 cycles of 3% dextran sulfate sodium salt (DSS, MP Biomedicals, Eschwege, Germany; MW, 36–50 kDa) given via the drinking water for 5 to 7 days. Due to the enhanced susceptibility of *H*. *polygyrus* infected mice to DSS treatment, these mice were treated with 2% DSS in the first cycle. Ten to twelve weeks after AOM injection, tumor distribution in the distal part of the colon was determined by murine colonoscopy [52], and tumor scores were calculated. In brief, mice were anesthetized by intraperitoneal injection of ketamine/xylazine, and a rigid endoscope (Coloview miniendoscopic system, Karl Storz, Tuttlingen, Germany) was inserted as far as possible into the rectum under visual control. Endoscopies were recorded while the endoscope was slowly withdrawn, starting at the flexure and stopping at the anus. Tumor sizes were graded on a scale of 1–5 according to the following grades: grade 1 (very small but detectable tumor), grade 2 (tumor covering up to one eighth of the colonic circumference), grade 3 (tumor covering up to a quarter of the colonic circumference), grade 4 (tumor covering up to half of the colonic circumference), and grade 5 (tumor covering more than half of the colonic circumference). Tumor scores per mouse were calculated by summing up the sizes/grades of all the tumors in a given mouse.

### Induction of DSS colitis and determination of clinical scores

Mice received 2% DSS (MP Biomedicals) in drinking water for 7 days. DSS was replaced by normal drinking water and mice were sacrificed one day later. Some of the mice were infected with 200 L3 *H*. *polygyrus* 6 days before DSS treatment. To determine disease activity indexes (DAI, 0–12) we used a validated scoring system; DAI consisted of loss of body weight (1, 1–5%; 2, 6–10%; 3, 11–15%; 4, 16–20%), rectal bleeding (0, no blood; 2, blood visible; 4, gross bleeding) and stool consistency (0, normal; 2, loose stool; 4, diarrhea)[53].

### Preparation of functionalized CaP-PLGA nanoparticles and application *in vivo*

CaP-PLGA Nanoparticles functionalized with siRNA directed against IL-6 and CXCL1 were prepared as described previously [54]. In brief, single shell nanoparticles were synthesized by fast mixing equal amounts of calcium-l-lactate and diammonium hydrogen phosphate. Instantly after mixing, the calcium phosphate dispersion was mixed with solutions of siRNA (4 mg mL^−^ 1, GE healthcare life sciences, Chalfont St. Giles, UK) to functionalize the particles. To encapsulate the calcium phosphate nanoparticles into the biodegradable polymer poly(d,l-lactide-*co*-glycolide) (PLGA, Resomer RG 502 H, Evonik Industries, Darmstadt, Germany), a water-in-oil-in-water double emulsion solvent evaporation method was applied. The dispersed polyvinylalcohol (PVA)-coated nanoparticles were shock-frozen in liquid nitrogen and lyophilized. Nanoparticles (8 μg of each siRNA per application) were administered intrarectally into mice sedated with low amounts of isoflurane using a small catheter. Mice were treated from day 1 onwards, after the start of 2% DSS treatment. The severity of colitis was assessed by disease activity index.

### *Heligmosomoides polygyrus* infection

*Heligmosomoides polygyrus* life cycle and production of larvae were conducted at the University of Edinburgh as described elsewhere [55]. BALB/c mice were infected with 200 third-stage (L3) larvae by oral gavage either at week 0 (6 days before the first DSS application) or at week 8 (6 days after the last DSS application) of CAC induction. Fecal egg counts were determined according to a standard protocol [56].

### Histology and immunohistochemistry of the colon and small intestine

Small intestines and colons were prepared and rinsed with PBS. Approximately 1 cm of the distal colon and the ileum were embedded in paraffin. Tissue sections of 4 μm were stained with hematoxylin and eosin (H&E) and periodic acid Schiff (PAS) to demonstrate mucopolysaccharides. Goblet cells were enumerated and referred to the length of the villi.

### Small intestine / Colon explant culture

After the preparation of the small intestines or colons, they were rinsed with PBS and cut open longitudinally. A small explant from the distal part of the colon or the ileum was taken and the weight was determined (around 10–20 mg). The biopsies were cultured for 6 h in 300 μl of RPMI (Invitrogen) supplemented with 10% heat-inactivated FCS, 25 mmol/L HEPES (both Biochrom, Berlin, Germany), 100 U/mL penicillin, and 0.1 mg/mL streptomycin (both Sigma-Aldrich) (complete media). Cytokine levels in the supernatants were measured by Polystyrene bead-based Luminex Assay (R&D Systems, Abingdon, UK). The assay was run on a Luminex 200 instrument. Cytokine concentrations were calculated using the Luminex IS software (Luminex Corporation, Austin, TX), and were normalized to the respective colon/ileum weight.

### Cell isolation and flow cytometry

Single-cell suspensions from mesenteric lymph nodes (mLNs) were prepared by meshing the lymph nodes through a 70 μm cell strainer and washing with PBS containing 2 mM EDTA and 2% FCS. Lamina propria lymphocytes (LPLs) were isolated as described previously [5]. In brief, tissue pieces of the colon were washed in PBS containing 3 mM EDTA. EDTA was removed by washing the tissue with RPMI containing 1% FCS, 1 mM EGTA, and 1.5 mM MgCl_2_. Single cells were obtained by digesting the tissue in RPMI containing 20% FCS and 100 U/mL collagenase (*Clostridium histolyticum*, Sigma-Aldrich, St. Louis, MO) at 37°C for 1 h with subsequent filtration through 40 μm and 30 μm cell strainer.

For flow cytometry analysis, single cells were incubated with fluorochrome-labeled antibodies against CD4 (RM4-5 or H129.19; BD Biosciences, Heidelberg, Germany), CD8 (53–6.7; BD Biosciences), CD62L (MEL-14; BD Biosciences), CD90.1 (OX-7; BD Biosciences), Ki67 (SolA15; eBioscience), IL-4 (11B11; BD Biosciences), and Foxp3 (FJK-16s; eBioscience). The Foxp3 staining kit from eBioscience was used according to the manufacturer‘s recommendations to stain Foxp3 and Ki67 intracellularly. To assess IL-4, cells were cultured for 4 h with 10 ng/mL PMA and 1 μg/mL ionomycin in the presence of 5 μg/mL Brefeldin A (all Sigma-Aldrich) in complete media. After surface staining, cells were fixed with 2% paraformaldehyde, permeabilized with 0.1% NP-40, and stained with antibodies against IL-4. Cells were acquired with a LSR II instrument and analyzed using DIVA software (both from BD Biosciences).

### Inhibition assay

CD4^+^Foxp3^+^ (eGFP^+^) Tregs were sort purified from mLNs of naïve Foxp3/eGFP reporter mice and Foxp3/eGFP mice infected with *H*. *polygyrus* for 10 days using a FACSAria II cell sorter (BD Biosciences). Using the CD4^+^ T-cell isolation kit II (Miltenyi Biotec, Bergisch-Gladbach, Germany), CD4^+^ responder T cells were enriched from spleens of naïve Thy1.1 mice. CD4^+^ responder T cells were labeled with eFluor670 (eBioscience). 1×10^5^ CD4^+^ responder T cells were either cultured alone or co-cultured with CD4^+^Foxp3^+^ (eGFP^+^) Tregs (1×10^5^) for 3 days in complete media in the presence of 1 μg/mL anti-CD3 (2C11; BD Biosciences) and irradiated splenocytes from naïve BALB/c mice, which served as antigen-presenting cells (APCs) (3×10^5^).

### Statistical analysis

Data were expressed as mean ± SEM. Results were tested for normal distribution using Kolmogorov-Smirnov or D'Agostino & Pearson omnibus normality test. Where appropriate, the unpaired Student *t* test or Mann Whitney test was used to compare two groups. Differences between means of more than two groups were assessed using one-way ANOVA followed by Tukey`s or Dunn's Multiple Comparison Test. Statistical significance between two groups at different time points was calculated using two-way ANOVA followed by Bonferroni posttests. Kaplan-Meier plots were used to analyze recovery of *H*. *polygyrus* infected mice. Comparisons of survival curves were made using the log-rank (Mantel-Cox) test. Statistical significance was set at the level of p<0.05. All analyses were calculated using the GraphPad Prism 7.03 software (La Jolla, CA).

## Supporting information

S1 Methods Induction of colitis in RAG2-/- and VILLIN-HA transgenic mice. (DOCX)

S1 Fig *H*. *polygyrus* infection decreases pro-inflammatory cytokine production in the small intestine. At day 1, mice were infected with 200 stage-three larvae (L3) *H*. *polygyrus* by oral gavage. Six days later, DSS was given via the drinking water for 7 days and mice were sacrificed on day 15. (A) Small intestines were prepared and representative tissue sections from DSS mice and DSS+*H*. *poly* mice were fixed and stained with hematoxylin and eosin (H&E) or periodic acid Schiff (PAS) to show pathologic changes. Images show magnification at x200. (B) Goblet cells in PAS stained sections were counted and referred to villi length. Bars represent the mean±SEM of data from one experiment (naïve, n = 2; naïve+*H*.*poly*, n = 3; DSS, n = 3; DSS+*H*.*poly*, n = 2). Statistical significance was calculated using one-way ANOVA followed by Tukey's Multiple Comparison Test (*, p≤ 0.05; **, p≤ 0.01). (C) Biopsies from small intestine samples were cultured *in vitro* for 6 hours in culture medium. Levels of IL-6 and CXCL1 in the supernatants were determined by Luminex. Bars show the mean ± SEM of cytokines per milligram tissue from 3 experiments (naïve, n = 10; naïve+*H*.*poly*, n = 12; DSS, n = 11; DSS+*H*.*poly*, n = 11). Statistical significance was calculated using one-way ANOVA followed by Dunn's Multiple Comparison Test (*, p≤ 0.05; **, p≤ 0.01).
(TIF)

S2 Fig *H*. *polygyrus* infection does not protect from RAG2^-/-^ and VILLIN-HA T cell transfer colitis. (A) Schematic time schedule of *H*. *polygyrus* (*H*.*poly*) infection and induction of T cell transfer colitis in RAG2^-/-^ mice. At week 0, RAG2^-/-^ mice were infected with 200 stage-three larvae (L3) *H*. *polygyrus* by oral gavage. Two weeks later, 5x10^5^ CD4^+^ CD45RB^hi^ cells were injected i.p. into RAG2^-/-^ mice or *H*.*poly* infected RAG2^-/-^ mice. At week 9 mice were sacrificed and the colons were prepared (B) Representative tissue sections of colon samples from RAG and RAG+*H*. *poly* mice were fixed and stained with hematoxylin and eosin (H&E) to show pathologic changes. Images show magnification at x200. Severity of colitis was assessed by scoring pathological changes. (C) Biopsies from colon samples were incubated *in vitro* for 6 hours in culture medium. Levels of IL-6 and CXCL1 in the supernatants were determined by Luminex and cytokines per milligram colon tissue were calculated. Graphs show the mean ± SEM of individual mice from 1 experiment (RAG, n = 4; RAG+*H*.*poly*, n = 4). (D) Schematic time schedule of *H*. *polygyrus* (*H*.*poly*) infection and induction of T cell transfer colitis in VILLIN-HA mice. At week 0, VILLIN-HA mice were infected with 200 stage-three larvae (L3) *H*. *polygyrus* by oral gavage. Two weeks later, 3x10^6^ HA-specific CD4^+^ Th1 polarized cells were injected i.v. into VILLIN-HA mice or *H*.*poly* infected VILLIN-HA mice. Five days after T cell transfer mice were sacrificed and the colons were prepared. (E) Representative tissue sections of colon samples from VILLIN-HA and VILLIN-HA+*H*. *poly* mice were fixed and stained with hematoxylin and eosin (H&E) to show pathologic changes. Images show magnification at x200. Severity of colitis was assessed by scoring pathological changes. Graphs show the mean ± SEM of individual mice from 1 experiment (VILLIN-HA, n = 4; VILLIN-HA+*H*.*poly*, n = 4).
(TIF)
