# Supplementary material for: Intestinal helminth infection drives carcinogenesis in colitis-associated colon cancer
Source: PLoS Pathog. 2017 Sep 22;13(9):e1006649. doi: 10.1371/journal.ppat.1006649 (PMC5627963; doi:10.1371/journal.ppat.1006649)
Supplement: S1 Methods — (DOCX) [file ppat.1006649.s001.docx]

**Supplemental Material and Methods:**

**Mice**

Female and male C.Tg(Tcra/Tcrb)1Vbo (termed TCR-HA) mice, C.Tg(Villin-HA/PR8/34)L2Gbf (termed VILLIN-HA) mice and C.Rag2tm1Fwa (termed RAG2^-/-^) mice were bred in-house and used at an age of six to ten weeks. VILLIN-HA transgenic mice express A/PR/34 influenza HA under the enterocyte-specific villin promoter [1]. TCR-HA transgenic mice possess CD4^+^ T cells expressing an α/β-TCR specific for the MHC class II H2E^d^:HA_110-120­_ -restricted epitope of the HA protein [2].

**Induction of colitis in RAG2^-/-^**

CD4^+^ T cells were enriched from spleens of female BALB/c mice by magnetic cell separation (CD4^+^ T cell isolation kit, Miltenyi Biotec, Bergisch Gladbach, Germany). CD4^+^ T cells were labeled with anti-CD4 (RM4-5, BD Biosciences) and anti-CD45RB (16A, Biolegend), and CD4^+^ CD45RB^hi^ cells were sorted using a FACSAria II cell sorter (BD Biosciences). RAG2^-/-^ mice were injected i.p. with 5x10^5^ CD4^+^ CD45RB^hi^ T cells. Two weeks prior to T cell transfer some of the RAG2^-/-^ mice were infected with 200 L3 *H. polygyrus.* At week 7 after T cell transfer mice were sacrificed and severity of colitis was scored by histopathology.

**Induction of colitis in VILLIN-HA transgenic mice**

HA-specific CD4^+^ T cells from the spleens of TCR-HA mice were sorted by flow cytometry and polarized into IFN-γ producing Th1 cells by adding plate-bound α-CD3 (5µg ml^-1^), soluble α-CD28 (1 µg ml^-1^) (both BD Biosciences), recombinant mouse IL-12 (20 ng ml^-1^, R&D Systems, Wiesbaden, Germany) and α-IL-4 antibody (200 ng ml^-1^, eBioscience, San Diego, CA) to the cell culture media. At day 6 the successful polarization was measured by staining for IFN-γ (XMG1.2, BD Biosciences) and flow cytometric analysis. Upon successful polarization 3 x 10^6^ HA-specific Th1 cells were adoptively transferred (i.v.) into VILLIN-HA transgenic mice. Mice were monitored daily for signs of sickness. Two weeks prior to CD4^+^ T cell transfer half of the mice were infected with 200 L3 *H. polygyrus.* Mice were sacrificed 5 days after T cell transfer and severity of colitis was scored by histopathology.

1. Westendorf AM, Templin M, Geffers R, Deppenmeier S, Gruber AD, Probst-Kepper M, et al. CD4+ T cell mediated intestinal immunity: chronic inflammation versus immune regulation. Gut. 2005;54(1):60-9.

2. Kirberg J, Baron A, Jakob S, Rolink A, Karjalainen K, von Boehmer H. Thymic selection of CD8+ single positive cells with a class II major histocompatibility complex-restricted receptor. J Exp Med. 1994;180(1):25-34.
