# Supplementary figures and images for: Intestinal helminth infection drives carcinogenesis in colitis-associated colon cancer
Source: PLoS Pathog. 2017 Sep 22;13(9):e1006649. doi: 10.1371/journal.ppat.1006649 (PMC5627963; doi:10.1371/journal.ppat.1006649)

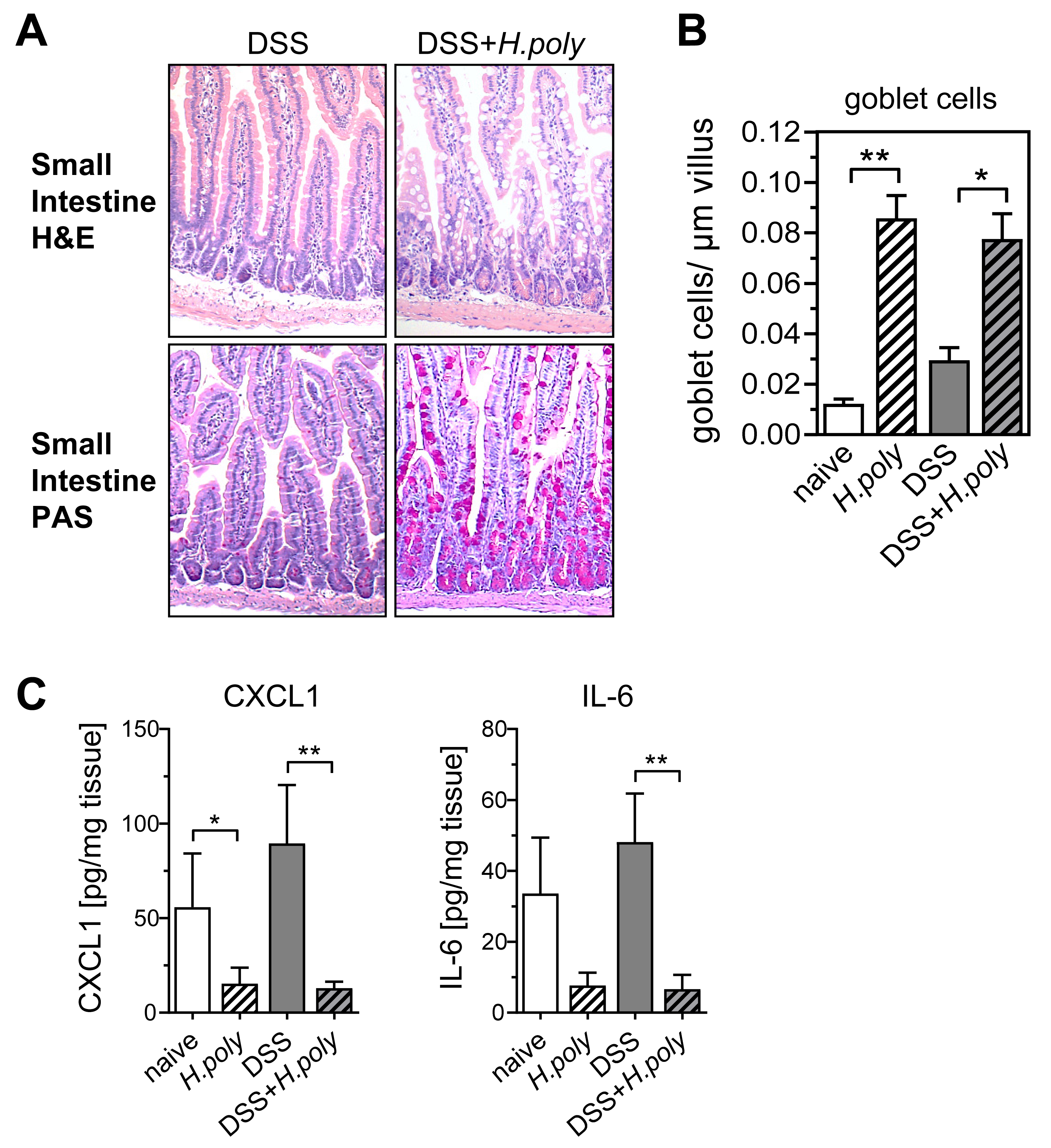

Supplement: S1 Fig — At day 1, mice were infected with 200 stage-three larvae (L3) H. polygyrus by oral gavage. Six days later, DSS was given via the drinking water for 7 days and mice were sacrificed on day 15. (A) Small intestines were prepared and representative tissue sections from DSS mice and DSS+H. poly mice were fixed and stained with hematoxylin and eosin (H&E) or periodic acid Schiff (PAS) to show pathologic changes. Images show magnification at x200. (B) Goblet cells in PAS stained sections were counted and referred to villi length. Bars represent the mean±SEM of data from one experiment (naïve, n = 2; naïve+H.poly, n = 3; DSS, n = 3; DSS+H.poly, n = 2). Statistical significance was calculated using one-way ANOVA followed by Tukey's Multiple Comparison Test (*, p≤ 0.05; **, p≤ 0.01). (C) Biopsies from small intestine samples were cultured in vitro for 6 hours in culture medium. Levels of IL-6 and CXCL1 in the supernatants were determined by Luminex. Bars show the mean ± SEM of cytokines per milligram tissue from 3 experiments (naïve, n = 10; naïve+H.poly, n = 12; DSS, n = 11; DSS+H.poly, n = 11). Statistical significance was calculated using one-way ANOVA followed by Dunn's Multiple Comparison Test (*, p≤ 0.05; **, p≤ 0.01). (TIF) [file ppat.1006649.s002.tif]

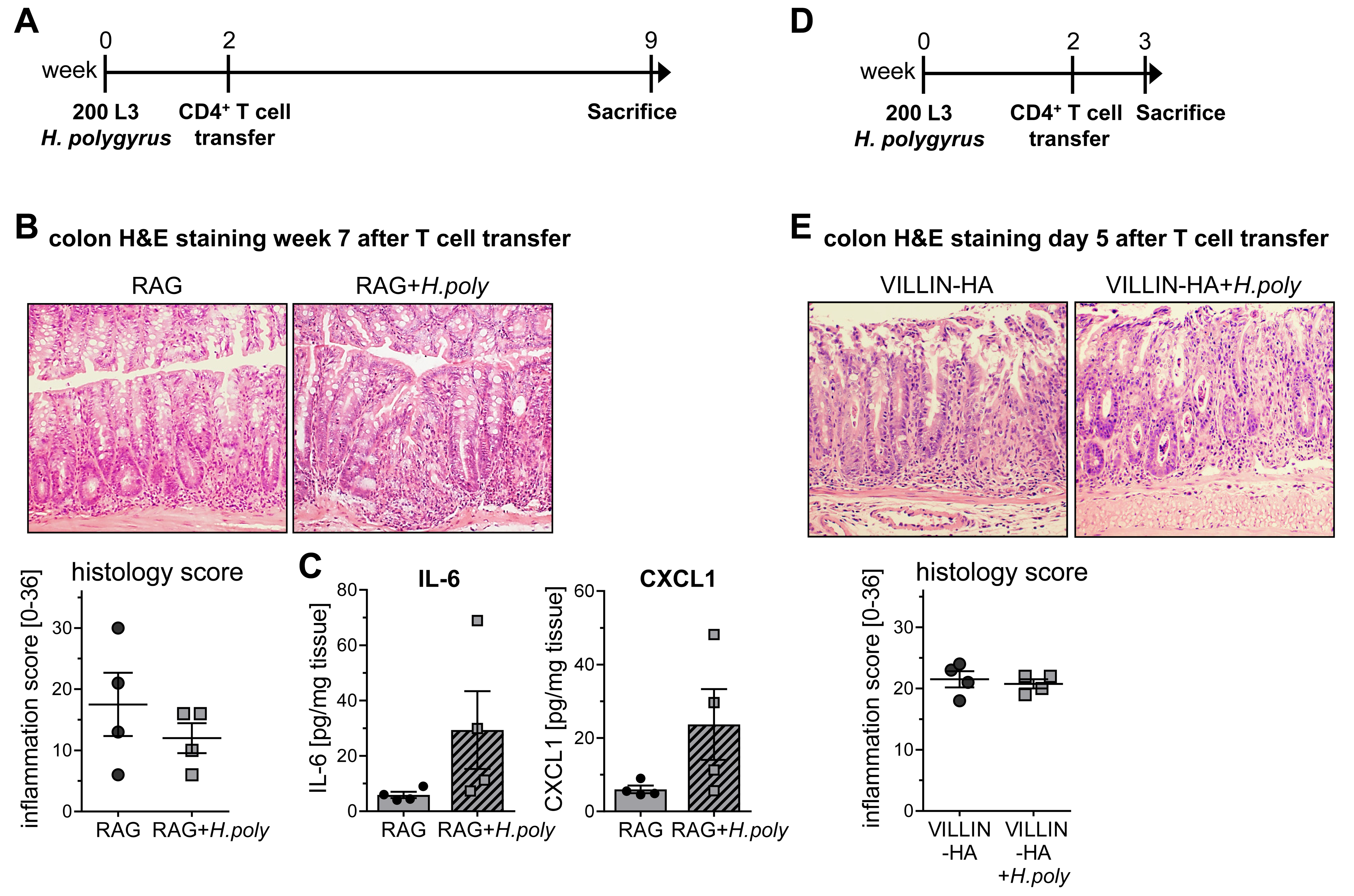

Supplement: S2 Fig — (A) Schematic time schedule of H. polygyrus (H.poly) infection and induction of T cell transfer colitis in RAG2-/- mice. At week 0, RAG2-/- mice were infected with 200 stage-three larvae (L3) H. polygyrus by oral gavage. Two weeks later, 5x10^5^ CD4+ CD45RBhi cells were injected i.p. into RAG2-/- mice or H.poly infected RAG2-/- mice. At week 9 mice were sacrificed and the colons were prepared (B) Representative tissue sections of colon samples from RAG and RAG+H. poly mice were fixed and stained with hematoxylin and eosin (H&E) to show pathologic changes. Images show magnification at x200. Severity of colitis was assessed by scoring pathological changes. (C) Biopsies from colon samples were incubated in vitro for 6 hours in culture medium. Levels of IL-6 and CXCL1 in the supernatants were determined by Luminex and cytokines per milligram colon tissue were calculated. Graphs show the mean ± SEM of individual mice from 1 experiment (RAG, n = 4; RAG+H.poly, n = 4). (D) Schematic time schedule of H. polygyrus (H.poly) infection and induction of T cell transfer colitis in VILLIN-HA mice. At week 0, VILLIN-HA mice were infected with 200 stage-three larvae (L3) H. polygyrus by oral gavage. Two weeks later, 3x10^6^ HA-specific CD4+ Th1 polarized cells were injected i.v. into VILLIN-HA mice or H.poly infected VILLIN-HA mice. Five days after T cell transfer mice were sacrificed and the colons were prepared. (E) Representative tissue sections of colon samples from VILLIN-HA and VILLIN-HA+H. poly mice were fixed and stained with hematoxylin and eosin (H&E) to show pathologic changes. Images show magnification at x200. Severity of colitis was assessed by scoring pathological changes. Graphs show the mean ± SEM of individual mice from 1 experiment (VILLIN-HA, n = 4; VILLIN-HA+H.poly, n = 4). (TIF) [file ppat.1006649.s003.tif]
